# A short-term efficacy comparison between the FNS and THA as interventions for unstable femoral neck fracture

**DOI:** 10.3389/fsurg.2025.1537335

**Published:** 2025-06-06

**Authors:** Kunpeng Si, Yuelei Zhang, Gang Wang

**Affiliations:** Department of Orthopaedics, First Affiliated Hospital of Anhui Medical University, Hefei, Anhui, China

**Keywords:** femoral neck fracture, femoral neck system, total hip arthroplasty, clinical effect, retrospective study

## Abstract

**Objectives:**

This study aimed to compare the difference in short-term clinical effects of internal fixation using the femoral neck system (FNS) and total hip arthroplasty (THA) on unstable femoral neck fractures.

**Methods:**

A retrospective analysis was conducted on 37 cases of unstable femoral neck fracture admitted to our hospital from 1 July 2020 to 30 June 2023. The cases were divided into an FNS group (21 cases) and a THA group (16 cases) based on the surgical method used. A comparative analysis was performed between the cohorts regarding demographic characteristics (sex and age), perioperative parameters (length of hospital stay, surgical duration, and intraoperative blood loss), and postoperative functional outcomes [Visual Analog Pain Score (VAS) and Harris Hip Score (HHS) assessments].

**Result:**

The operative time, length of hospital stay, and intraoperative blood loss in the FNS group were significantly lower than those in the THA group, and the time of weight bearing on the ground in the FNS group was significantly longer than that in the THA group (*P* < 0.01). The comparative analysis revealed comparable outcomes in postoperative pain intensity (VAS) and functional recovery (HHS) between the cohorts, with both parameters demonstrating statistically equivalent values (*P* > 0.05).

**Conclusion:**

For unstable femoral neck fractures, FNS and THA were equally effective. Although shortening and necrosis were observed in the FNS group, no statistically significant difference in postoperative complications was found between the two groups. The operative time of FNS was shorter, with less intraoperative bleeding. However, the earlier weight-bearing time of THA was more conducive to the recovery of limb function.

## Introduction

Femoral neck fractures constitute approximately 56% of total hip fracture occurrences among the geriatric demographic (1). The etiology is often caused by low-energy damage, such as that from falls. Among them, unstable femoral neck fractures are prone to secondary displacement because of their strong shearing force at the fracture end, which leads to further damage to the blood supply. In general, surgery is needed. However, there is still no unified opinion on treating unstable femoral neck fractures.

For young and middle-aged patients who are in good health, to obtain better hip function, internal fixation can be given priority. Even in the presence of late-stage complications, including femoral head necrosis, revision total hip arthroplasty (THA) maintains its efficacy in achieving favorable mid- to long-term clinical outcomes (2). Nevertheless, internal fixation for unstable femoral neck fractures continues to demonstrate elevated rates of postoperative complications, including non-union, femoral neck shortening, and avascular necrosis of the femoral head (AVN) (3). Particularly in cases that develop post-fixation osteonecrosis, secondary total hip arthroplasty remains essential for functional restoration. Thus, for the 55–70 age cohort, existing research evidence suggests superior clinical outcomes with total hip arthroplasty compared to internal fixation in the management of unstable femoral neck fractures (4).

As a novel therapeutic approach for managing unstable femoral neck fractures, the femoral neck system (FNS) has strong anti-shear force and anti-rotation ability at the fracture end and can provide stable support. Biomechanical analysis also confirms that FNS has certain advantages compared with traditional implants, such as compression cannulated screw (CCS) and dynamic hip screw (DHS). For example, the average axial stiffness of FNS is 748.9 ± 211.4 N/mm > DHS 688.8 ± 132.6 N/mm > CCS 584.1 ± 156.6 N/mm, and can reduce the incidence of complications such as femoral neck shortening and femoral head necrosis after operation (5). In this study, the short-term effects of FNS and THA in patients with unstable femoral neck fractures were analyzed retrospectively for clinical reference.

## Patients and methods

### Patients

Informed consent was exempted as this retrospective analysis used de-identified data without compromising patient confidentiality.

A retrospective analysis was performed on patients diagnosed with unstable femoral neck fractures who were admitted to the First Affiliated Hospital of Anhui Medical University during the period from 1 July 2020 to 30 June 2023. Inclusion criteria were as follows: (1) age 50–60 years; (2) fresh femoral neck fracture with definite diagnosis; (3) fracture classification conforms to Pauwels III type, and Garden III or IV femoral neck fractures; and (4) can walk freely before fracture. The exclusion criteria were as follows: (1) fracture of the hip and other parts of the legs, (2) pathological fracture, or (3) incomplete information. According to the one-stage operation, 21 patients were divided into the FNS group, namely 6 men and 15 women, ranging in age from 50 to 59 years, with an average age of 54.86 ± 2.68 years. Furthermore, there were 16 cases who underwent THA, namely, 6 men and 10 women, ranging in age from 52 to 60 years, with an average age of 56.75 ± 2.33 years (Table 1). Considering that there was a significant age difference between the two groups (*P* = 0.035 < 0.05), a regression analysis was used to calculate the age difference to evaluate its impact on the observation indicators (Table 2). The results show that the age factor had no significant effect on the observation indices, with *P* > 0.05 for all.

**Table 1:** Comparison of sex, age, and preoperative Garden classification between the two groups.

Group	Number	Sex		Age (years)	Garden classification before operation	
		Male	Female		Type III	Type IV
FNS	21	6	15	54.86 ± 2.68	11	10
THA	16	6	10	56.75 ± 2.33	3	13
*t*/*χ*^2^		0.33		−2.189	4.367	
*P*		0.565		0.035	0.037	

**Table 2:** Analysis of the degree of influence of the age factor on observation indicators.

Independent variable	Observation indicator	B	*P*
Age (years)	Length of hospital stay （days）	0.247	0.167
	Operative time (min)	1.951	0.351
	Intraoperative blood loss （ml）	8.678	0.064
	Postoperative 2-day VAS	−0.016	0.734
	Postoperative 1-month VAS	0.051	0.334
	HHS at the 3-month postoperative follow-up	0.148	0.226
	HHS at the 6-month postoperative follow-up	0.048	0.717
	Incidence rate of complications	0.380	0.193

### Research and surgical method

Routine preoperative preparation was completed for all patients, which included comprehensive preoperative hip imaging assessments and the exclusion of surgical contraindications. Preoperative assessments were systematically performed utilizing the American Society of Anesthesiologists (ASA) Physical Status Classification System, with subsequent anesthetic management uniformly implemented by a specialized anesthesia team. The surgeries were performed by the same treatment team. Anticoagulation therapy with low molecular weight heparin was initiated preoperatively, while infection prophylaxis was achieved through intravenous administration of first-generation cephalosporins (e.g., cefazolin) 0.5–1 h prior to surgical incision.

In the FNS group, following anesthesia induction, the patient was positioned supine with their lower limbs stabilized by a traction apparatus, followed by hip flexion, abduction, and internal rotation adjustments. Preliminary fracture reduction was confirmed under C-arm fluoroscopic guidance, after which routine surgical disinfection and sterile draping were completed. A 4–5 cm longitudinal incision parallel to the femoral shaft axis was created at the femoral trochanteric level and layered dissection was performed to expose the inferior trochanteric region. Under fluoroscopic control, Kirschner wires were inserted across the fracture line along the femoral neck axis for provisional fixation, followed by FNS guide assembly placement and guidewire advancement along the femoral neck axis. Fluoroscopic verification confirmed a 5 mm distance between the guidewire tip and femoral head subchondral bone. Reaming was performed along the guidewire using a cannulated reamer, after which an appropriately sized FNS sleeve and power rod assembly were positioned. Upon confirmation of satisfactory fluoroscopic alignment, anti-rotation screws and locking screws were sequentially inserted via the guide system. Fracture compression was applied post-adjustment, and the incision was irrigated and closed in layers following final radiographic confirmation of the reduction quality.

In the THA group, following anesthesia administration, the patient was positioned laterally with pelvic stabilization achieved using a fixation frame. A posterolateral hip approach was utilized, beginning with an 8-cm posterior incision extending medially from the superior border of the femoral greater trochanter to the anterior inferior iliac spine. This was combined with a 7-cm distal incision parallel to the femoral shaft axis, resulting in a total 15-cm surgical exposure. The hip joint capsule was sequentially dissected to expose the femoral neck fracture, after which the femoral head and fracture fragments were excised. The acetabular fossa was prepared through soft tissue debridement followed by progressive acetabular reaming until subchondral bleeding was observed. An appropriately sized acetabular component was press-fit into the prepared cavity, and a corresponding liner was secured. The femoral neck osteotomy site was then exposed, and the medullary canal was sequentially reamed. A trial femoral stem prosthesis was inserted, and hip joint reduction was performed to assess stability. Upon achieving a satisfactory range of motion and stability, the trial components were replaced with permanent prostheses with identical dimensions. Final intraoperative assessment of joint biomechanics was conducted, after which the surgical site was irrigated and closed in anatomical layers.

### Postoperative treatment

All the patients were given anti-infection prophylaxis with first-generation cephalosporin for 24 h after operation, and enoxaparin was routinely used to prevent venous thrombosis in the lower limbs 12 h after the operation. In the FNS group, strength training of the lower limb muscle pump was started in bed the day after the operation to help in the recovery of limb function. Early functional exercise was carried out 1–2 weeks later, and ambulation time was strictly controlled. The weight-bearing time under partial load started at 1 h/day and gradually increased. In the THA group, on the second day after operation, with the help of a walker, the patient moved under partial weight-bearing (PWB). The average time taken for partial weight-bearing ambulation with the assistance of a walking aid after THA surgery was 1.188 ± 0.390 days (some patients were able to partially ambulate on the second day after surgery after adjusting the personalized analgesia plan). The patients in both groups continued to receive oral aspirin to prevent deep venous thrombosis in the lower limbs after discharge. After the patient was discharged from the hospital, a hip x-ray was conducted and examined at scheduled time points of 1, 3, 6, and 12 months.

### Perioperative-related indicators and follow-up observation index

The perioperative observation indicators were recorded, including length of hospital stay, operative time, intraoperative blood loss, and 2-day postoperative Visual Analog Pain Score (VAS). Postoperative follow-up metrics were systematically assessed, comprising VAS at 1-month intervals and Harris Hip Score (HHS) evaluations conducted at 3- and 6-month postoperative intervals. Procedure-related complications were radiographically defined as either AVN or femoral neck shortening. AVN was characterized by cortical step-off deformity (>5° angulation), bicortical parallel line signs at the femoral base, and morphological collapse of the femoral head architecture. Femoral neck shortening was quantitatively confirmed when radiographic measurements demonstrated >5 mm reduction in neck length compared to preoperative baselines. Other complications encompassed surgical site infections, neurovascular injuries, and lower extremity deep vein thrombosis; however, none of these adverse events were observed throughout the study's follow-up period.

### Statistical method

All the statistical analyses were performed using SPSS version 27.0. The normality of data distribution was assessed using the Shapiro–Wilk test. Normally distributed continuous variables were expressed as mean ± standard deviation (x¯ ± s) and analyzed using an independent samples *t*-test. Non-normally distributed data were presented as median (interquartile range) [M (P25–P75)] and compared using the Mann–Whitney *U*-test. Categorical variables were analyzed by a χ^2^ test when all expected counts were ≥5; when at least two expected counts were <5, Fisher's exact test was utilized. Relationships between variables were assessed using Pearson correlation analysis. A significance threshold of *P* < 0.05 was applied for all statistical tests.

## Result

A minimum 12-month follow-up period was maintained for all enrolled patients. Significantly shorter operative time (*P* = 0.001) and reduced intraoperative blood loss (*P* = 0.001) were observed in the FNS group compared to the THA group (*P* < 0.05). The comparative analysis of postoperative outcomes revealed no significant intergroup differences in pain assessment (VAS) and functional recovery (HHS) scores across follow-up evaluations, with all comparative results demonstrating statistical equivalence (VAS: *P* = 0.141, 0.411; HHS: *P* = 0.471, 0.099, 0.204; all *P* > 0.05). The Garden classifications of the preoperative fractures in the two treatment groups were Garden III or IV, and there was no significant difference in the HHS between those classified as Garden III and IV at 3 months after surgery (*P* = 0.054 > 0.05) and 6 months after surgery (*P* = 0.123 > 0.05). Furthermore, the FNS group's Garden alignment index grade was grade I or II, and the FNS group was classified according to the reduction quality. There was no significant difference in the HHS at 3 months (*P* = 0.550 > 0.05) and at 6 months after surgery (*P* = 0.925 > 0.05). In the FNS cohort, one case of femoral head avascular necrosis and one case of femoral neck varus deformity (angulation >15°) were radiographically confirmed, but there were no complications such as lower extremity deep vein thrombosis, non-union of femoral neck fracture, infection, and neurovascular injury. In contrast, no postoperative complications, including surgical site infection, delayed wound healing, or prosthetic dislocation, were documented in the THA group throughout the observation period.

**Figure 1:**
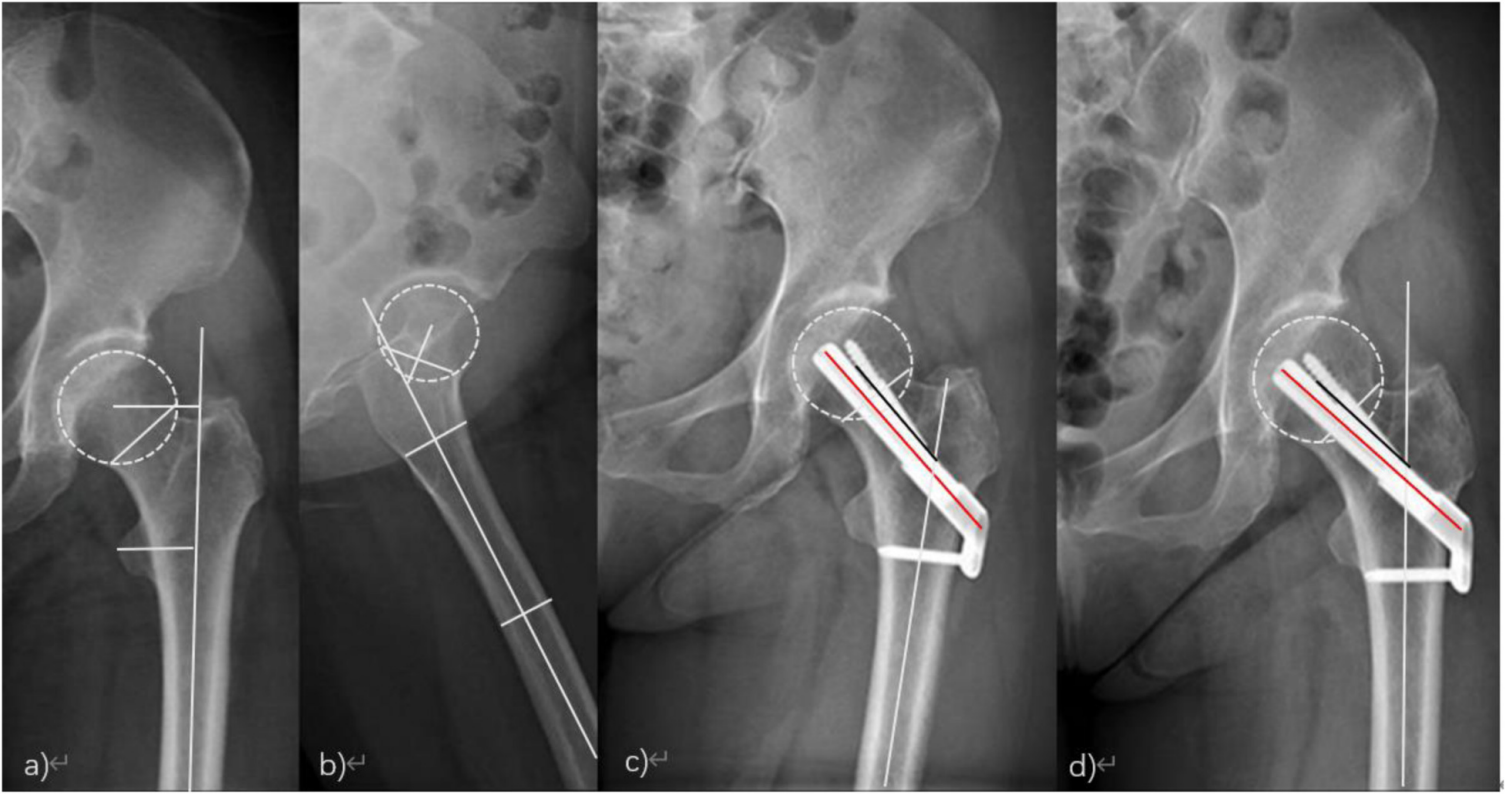
**(a**,**b)** Anterior hip position, femoral neck cortical discontinuity, and no obvious fracture displacement; **(c)** good alignment of femoral neck fracture and stable internal fixation 3 months after the operation; **(d)** the internal fixation was stable, the fracture line disappeared, and there was no femoral neck shortening or necrosis 6 months after the operation.

In case 1, a typical case, a 50-year-old woman was treated as “a fall caused left hip pain with limited activity for 3 days”. Upon admission, the physical examination found that the left lower limb was deformed with decreased external rotation of approximately 60°. The left hip was widely tender, with axial tapping pain (+), and it was difficult for the patient to participate in activities. An emergency hip x-ray showed a fracture of the left femoral neck. It was diagnosed as a fracture of the femoral neck (Garden III), and FNS internal fixation was performed after the surgical contraindications were ruled out. The preoperative and postoperative imaging data are shown in Figure 1.

**Figure 2:**
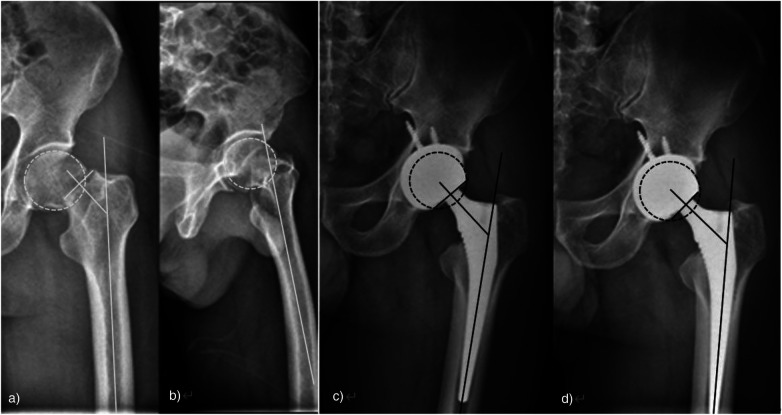
**(a**,**b)** Anterior position of hip joint and obvious displacement of femoral neck fracture; **(c)** orthopelvic position 3 months after THA; **(d)** the pelvis was in the right position and the prosthesis was stable in position 6 months after the operation.

In case 2, another typical case, a 56-year-old man was treated as “a car crash caused the left hip pain with limited activity for 2 days.” Upon admission, the physical examination showed that the left lower limb was 1.5 cm shorter than the right lower limb, showing external rotation deformity, obvious tenderness of the left hip, and insufficient movement. An emergency hip x-ray examination showed that the continuity of the left femoral neck was interrupted and the displacement was obvious. It was diagnosed as a fracture of the femoral neck (Garden IV). THA was performed after the surgical contraindications were ruled out. The preoperative and postoperative imaging data are shown in Figure 2.

## Discussion

Femoral neck fractures remain a prevalent clinical entity within geriatric trauma populations and emerging epidemiological data demonstrate a rising incidence among younger demographics, particularly in association with high-energy trauma mechanisms (6). Surgical management options are primarily divided into internal fixation systems and THA. Internal fixation modalities include cannulated screws, DHSs, medial buttress plates, and the FNS (7, 8). The FNS described in this study integrates multiple biomechanical advantages through optimized design parameters (9, 10). The design of the 130° angle between the power rod and the lateral locking plate is closest to the size of the physiological femoral neck-shaft angle in the Asian population. Thus, it can effectively disperse the vertical shear stress of the fractured end and convert the shear force into compressive stress along the direction of the power rod. It not only provides angular stability for the fractured end and prevents the femoral neck from turning over but also provides sliding pressure for the fractured end to promote the healing of the fractured end. In addition, the anti-rotation screw in the FNS device forms a plane fixation with the power rod and fixes the femoral head through mechanical locking. The two jointly share the stress of the femoral neck part of the FNS, which not only reduces the risk of fracture due to the local stress concentration of the internal fixation but also stops the fractured end from rotating. The locking screw of the FNS device is fixed to the femur by multi-axial support through the lateral plate together with the power rod and the anti-rotation screw, which enhances the overall biomechanical stability.

The comparative analysis demonstrated significantly reduced operative time and decreased intraoperative blood loss in the FNS cohort compared to the THA group, with both parameters showing statistically significant differences (*P* < 0.01 < 0.05) (Table 3). The implementation of a standardized postoperative pain management protocol resulted in equivalent pain assessment outcomes between the cohorts, with VAS demonstrating no statistically significant differences at both the 2-day (*P* = 0.141) and 1-month (*P* = 0.411) postoperative intervals (all *P* > 0.05). The comparative analysis of functional recovery outcomes revealed comparable Harris Hip Scores in the THA and FNS cohorts at both the 3-month (*P* = 0.471) and 6-month (*P* = 0.099) postoperative evaluations, with neither timepoint demonstrating statistical significance (both *P* > 0.05) (Table 4).

**Table 3:** Comparison of the perioperative indicators between the FNS group and the THA group.

Group	Number	Length of hospital stay （days）	Operative time (min)	Intraoperative blood loss （ml）	Postoperative 2-day VAS
FNS	21	5.95 ± 2.09	53.33 ± 9.40	52.57 ± 14.02	3.143 ± 0.91
THA	16	9.88 ± 2.31	100.94 ± 35.60	202.5 ± 21.13	3.5 ± 0.52
*t*		−5.416	−5.212	−24.555	−1.508
*P*		0.001	0.001	0.001	0.141

**Table 4:** Comparison of the follow-up indicators between the FNS group and the THA group.

Group	Numbers	VAS at the 1-month postoperative follow-up	HHS at the 3-month postoperative follow-up	HHS at the 6-month postoperative follow-up	Was there any complication? （Fisher's exact test）	
					Yes	No
FNS	21	1.95 ± 0.81	89.05 ± 2.29	93.67 ± 2.52	2	19
THA	16	2.19 ± 0.91	88.56 ± 1.55	92.50 ± 1.27	0	16
*t*/*χ*^2^		−0.832	0.729	1.694		
*P*		0.411	0.471	0.099	0.495	

Regarding postoperative rehabilitation, partial weight-bearing exercises could be initiated on the first postoperative day in the THA group, whereas strict bed rest was mandated for the FNS fixation group during the early recovery phase. This prolonged immobilization was associated with increased risks of hypostatic pneumonia, pressure ulcers, and lower extremity deep vein thrombosis (11). Beyond these immediate complications, long-term complications related to FNS fixation were found to significantly influence fracture healing outcomes. It has been reported in clinical studies (12) that mechanical complications, including screw breakage, cut-out, and loosening, occurred in 18.79% of cases when treating unstable femoral neck fractures with primary FNS fixation, ultimately leading to fracture displacement or discontinuity through biomechanical failure. Of particular concern was the development of AVN, the most severe long-term complication following FNS procedures. During early-stage AVN (Ficat I-II), characteristic low-intensity signals on T1-weighted MRI sequences were observed while femoral head integrity was maintained without collapse. For younger patients and functionally demanding elderly individuals, joint-preserving strategies such as core decompression, vascularized bone grafting, biological agents, or stem cell therapies were demonstrated to achieve preservation success rates exceeding 50% (13). However, when AVN progressed to advanced stages (Ficat III–IV), structural collapse of the femoral head was shown to result in severe hip dysfunction, necessitating subsequent THA intervention.

Risk factor analysis revealed that Garden IV fractures, femoral neck tilt angles >15°, suboptimal fracture reduction, and diabetes mellitus were independently associated with early AVN development post-FNS (14, 15). Finite element biomechanical analyses (16) further demonstrated that deviations from the standard 10° femoral neck tilt angle were correlated with progressive increases in femoral head stress concentration. This pathomechanical relationship between abnormal stress distribution and AVN progression was subsequently validated in clinical investigations (17).

For patients with unstable femoral neck fractures who are treated with THA for the first time, an unavoidable situation is the problem of multiple renovations caused by the limited service life of artificial joints, especially for relatively young patients, some of whom still have to participate in the workplace and life. The amount of activity and the degree of wear and tear of the prosthesis are high, so they are more likely to face the problem of multiple renovations of the prosthesis. According to relevant studies, the postoperative 10-year revision rate of THA for femoral neck fractures in young patients (<50 years old) is approximately 20%–30%. However, relevant research statistics also show that more than 90% of currently used artificial hip prostheses have a lifespan of more than 14 years, and some prostheses even have a lifespan of more than 30 years.

In comparison, the proportion of THA revision 10 years after surgery in young patients with femoral neck fractures undergoing initial FNS internal fixation was 25%–35%, slightly higher than that in the first-time THA population. Young patients who undergo multiple hip revision surgeries will not only have an increased risk of infection, but also hip bone loss and scarring of surrounding tissues. This increases the risk of prosthetic failure in the short term due to artificial joint dislocation, periprosthetic fractures, and aseptic loosening of the prosthesis, which not only affects the recovery of hip joint function but also causes tremendous psychological pressure for patients.

Combined with the above research, we think that when deciding whether to adopt FNS or THA in the first-stage operation of an unstable femoral neck fracture, we should comprehensively consider the following factors: (1) the functional level of the hip joint and the quality of life requirements of the patient before injury; (2) whether there are systemic diseases in the patient’s history, as diabetes, especially, is not well-controlled; (3) preoperative imaging examination, i.e., whether the fracture type is Garden IV or femoral neck anteversion > 15°; and (4) whether the expected fracture can be well reduced. The choice of surgical method is not only a technical decision, but also requires the patient's participation. The surgeon needs to have a full understanding of the patient's past life conditions. At the same time, preoperative hip x-rays and further CT examinations can more clearly identify the patient's fracture type and the physiological and anatomical characteristics of the femoral neck. The surgeon can then predict the degree of femoral neck injury and the difficulty of reduction during surgery with greater confidence. For patients with comminuted fractures of the posterior lateral femoral neck, THA treatment can be considered in the first phase. If it is a simple fracture of the femoral neck and the posterior lateral wall of the femoral neck is relatively complete, FNS fixation can be considered in the first phase, which is better for some younger patients and older patients in better physical condition (18, 19). This study was a single-center retrospective study, with a small sample size, and a large-sample multi-center randomized controlled study would provide more definitive results. The higher proportion of Garden IV fractures in the THA group may reflect clinical preferences for arthroplasty in severe displacement cases, potentially influencing necrosis rates. Future studies should stratify by fracture subtype to mitigate this bias. In this study, there were also significant differences in age distribution between the two groups of patients, but the age difference did not have a significant impact on the observation indicators. In addition, the overall follow-up time of this study was short, making it difficult to fully evaluate long-term hip function recovery and differences in quality of life. The conclusions may have inevitable statistical errors and contingency, and thus are for reference only. In future research, we will increase the generalizability of the research conclusions by conducting multi-center randomized trials in multiple medical centers and increasing the number of cases included. An extended follow-up will be conducted to observe differences in the long-term quality of life of patients who receive the two surgical procedures.

## Conclusion

For patients with unstable femoral neck fracture aged 50–60 years, if the patient's quality of life before injury is high and after the evaluation of the patient’s physical condition and preoperative hip imaging examination and the exclusion of high-risk factors such as AVN after internal fixation, FNS internal fixation can obtain a better prognosis for hip joint function. This treatment approach demonstrates particular suitability for younger individuals within this demographic range and senior patients exhibiting a robust physical status and elevated functional demands for hip joint performance. Even if adverse complications such as AVN still occur in the later stages and revision surgery is needed, such patients still have good tolerance and hip joint function level after revision. However, when the patients’ quality of life is poor before injury and their self-care is limited, especially for those who have systemic diseases such as diabetes, there are high-risk factors in preoperative hip imaging examination that lead to adverse complications such as AVN after internal fixation. THA treatment in the first stage allows the patient’s hip joint function to recover quickly, reduces bed rest time, and then reduces the number of related complications. During the clinical diagnosis and treatment process, clinicians aim to provide the best solution they can think of, analyze the advantages and disadvantages, and strive for excellence in surgical techniques. Patients have full choice after fully understanding the relevant risks; if their choice is not the recommended treatment, we should fully respect and consider it.

## Data Availability

The raw data supporting the conclusions of this article will be made available by the authors, without undue reservation.
